# Patient perspectives of artificial intelligence as a medical device in a skin cancer pathway

**DOI:** 10.3389/fmed.2023.1259595

**Published:** 2023-11-16

**Authors:** Anusuya Kawsar, Khawar Hussain, Dilraj Kalsi, Polychronis Kemos, Helen Marsden, Lucy Thomas

**Affiliations:** ^1^Chelsea and Westminster Hospital NHS Foundation Trust, London, United Kingdom; ^2^Skin Analytics Ltd., London, United Kingdom; ^3^Blizard Institute, Queen Mary University of London, London, United Kingdom

**Keywords:** artificial intelligence, skin cancer, dermatology, patient perspectives, medical device, AI as a medical device, deep ensemble for the recognition of malignancy (DERM), Skin Analytics

## Abstract

The use of artificial intelligence as a medical device (AIaMD) in healthcare systems is increasing rapidly. In dermatology, this has been accelerated in response to increasing skin cancer referral rates, workforce shortages and backlog generated by the COVID-19 pandemic. Evidence regarding patient perspectives of AIaMD is currently lacking in the literature. Patient acceptability is fundamental if this novel technology is to be effectively integrated into care pathways and patients must be confident that it is implemented safely, legally, and ethically. A prospective, single-center, single-arm, masked, non-inferiority, adaptive, group sequential design trial, recruited patients referred to a teledermatology cancer pathway. AIaMD assessment of dermoscopic images were compared with clinical or histological diagnosis, to assess performance (NCT04123678). Participants completed an online questionnaire to evaluate their views regarding use of AIaMD in the skin cancer pathway. Two hundred and sixty eight responses were received between February 2020 and August 2021. The majority of respondents were female (57.5%), ranged in age between 18 and 93 years old, Fitzpatrick type I-II skin (81.3%) and all 6 skin types were represented. Overall, there was a positive sentiment regarding potential use of AIaMD in skin cancer pathways. The majority of respondents felt confident in computers being used to help doctors diagnose and formulate management plans (median = 70; interquartile range (IQR) = 50–95) and as a support tool for general practitioners when assessing skin lesions (median = 85; IQR = 65–100). Respondents were comfortable having their photographs taken with a mobile phone device (median = 95; IQR = 70–100), which is similar to other studies assessing patient acceptability of teledermatology services. To the best of our knowledge, this is the first comprehensive study evaluating patient perspectives of AIaMD in skin cancer pathways in the UK. Patient involvement is essential for the development and implementation of new technologies. Continued end-user feedback will allow refinement of services to ensure patient acceptability. This study demonstrates patient acceptability of the use of AIaMD in both primary and secondary care settings.

## Introduction

The use of artificial intelligence (AI) is currently being explored across the field of medicine and the deployment of AI as a medical device (AIaMD) in healthcare systems is rapidly expanding. In dermatology, this has been accelerated in response to increasing skin cancer referral rates, workforce shortages and backlog generated by the COVID-19 pandemic (1). AI is making significant contributions in dermatology especially with automated skin lesion analysis, triage of cutaneous lesions, skin cancer detection, and dermatological image recognition, offering benefits to enhance various aspects of patient care (2). However while AI holds great potential, it is not without its challenges with potential concerns regarding patient data privacy and confidentiality, while ensuring these technologies are validated, reliable and accurate (3).

The deep ensemble for recognition of malignancy (DERM) device, designed by Skin Analytics, is an AIaMD that analyzes images of skin lesions to support the identification and appropriate management of skin cancers, premalignant lesions, and benign conditions. DERM was the first UKCA Class IIa certified AIaMD dermatology device on the UK market (4).

Evidence regarding patient perspectives of AIaMD being used by doctors to help make decisions about their care, is currently lacking in the literature. Patient acceptability is fundamental if this novel technology is to be effectively integrated into care pathways and patients must be confident that it is implemented safely, legally, and ethically.

The aim of this study was to explore patients’ perspectives on the use of AI as part of their skin cancer management pathway.

## Methods

A prospective, single-center, single-arm, masked, non-inferiority, adaptive, group sequential design trial, designed to demonstrate the potential of DERM to reduce unnecessary referrals, was conducted at Chelsea and Westminster Hospital in London, UK (ClinicalTrials.gov: NCT04123678). Patients over the age of 18, who were referred to a teledermatology skin cancer clinic with at least one skin lesion that could be photographed, were eligible for the study. Patients provided written informed consent for the study, and there was no financial compensation. Ethical approval for the study was granted by the West Midlands, Edgbaston Research Ethics Committee.

Patients attended an appointment with a clinical photographer based within the hospital. In addition to images of the lesions captured for standard of care assessment, macroscopic and dermoscopic images of each skin lesion were taken by a healthcare assistant using an iPhone X smartphone with Dermlite DL1 basic dermoscopic lens attachment. Captured images were uploaded for analysis by DERM, which was certified as a Class I AIaMD at the time. The DERM analysis result was not shared with the patient or the dermatologist, and the patient’s care continued in accordance with routine standard of care. Information on lesion history, risk factors for skin cancer, number of appointments needed to diagnose, and the final diagnosis were collected (5).

After their assessment participants were sent a link by email to an online questionnaire (Supplementary material) which was designed to evaluate their views regarding potential use of AIaMD in the skin cancer pathway. The questionnaire was hosted on an electronic Case Report Form that could be linked with the study record. Reminders were sent to patients who had not completed the survey after at least 1 week. The questionnaire included 4 questions on healthcare appointments prior to their teledermatology appointment, and 14 questions that evaluated patient acceptance of: (i) clinic and photography appointments (ii) AI as a service tool, which were worded both positively and negatively to minimize bias. A visual analog scale (VAS) was used to assess respondents’ satisfaction. The VAS ranged from 0 to 100 with a score of >50 taken to indicate an agreement with a given statement. The impact of patient factors (age, sex, and fitzpatrick skin type) and management outcome on the patient’s response were evaluated using a Kruskal-Wallis (KW) test, with statistical significance set at *p* < 0.05. Statistical analysis was conducted using the R language version 4.1.3 and environment for statistical computing.

## Results

Seven hundred patients were recruited between February 2020 and August 2021, including 12 patients who consented twice. Two hundred and sixty eight questionnaire responses were received (38.2% response rate), including two patients who completed the questionnaire twice. Respondents ranged in age between 18 and 93 years old. Most respondents were female (*n* = 154, 57.5%) and had Fitzpatrick type I-II skin (*n* = 218, 81.3%); however all 6 skin types were represented (Table 1).

**Table 1:** Baseline characteristics of study population.

	Respondents (*N*, %) Total *n* = 268
**Gender**	
Male	114 (43%)
Female	154 (57%)
**Age groups**	
18–29 years	35 (13%)
30–39 years	33 (12%)
40–49 years	28 (10%)
50–59 years	36 (13%)
60–69 years	50 (19%)
70–79 years	62 (23%)
80+	24 (9%)
**Fitzpatrick skin type**	
I	72 (27%)
II	146 (54%)
III	44 (16%)
IV	3 (1%)
V	1 (0.3%)
VI	2 (0.7%)
**Management outcome**	
Discharge	83 (31%)
Routine appointment	66 (25%)
Biopsy/urgent follow up	118 (44%)

Most patients (*n* = 207, 77.5%) attended the teledermatology clinic within 14 days of their GP appointment and reported that this time was “about right” for them (Table 2). Most patients (*n* = 191, 71.3%) reported never, or only once or twice, visiting a doctor about the same skin lesions in the past 5 years, with the median number of prior healthcare appointments, to assess the skin lesion/s included in the study, being 1 (IQR = 1–1, max 6).

**Table 2:** Appointments made by respondents prior to attending teledermatology clinic.

Question	Option	Respondents (*N*, %)
Number of visits to GP about lesions on my skin, over the last 5 years	Never	73 (27%)
	Once or twice	118 (44%)
	A couple of times	38 (14%)
	Several times	31 (12%)
	Quite a lot	6 (2%)
Number of days since my GP appointment	2 days	5 (2%)
	5 days	24 (9%)
	7 days	42 (16%)
	14 days	136 (51%)
	28 days	27 (10%)
	More than 28 days	24 (9%)
The time between seeing the GP and attending the teledermatology clinic was…	Too short	3 (1%)
	About right	224 (84%)
	Too long	20 (7%)
	Far too long	10 (4%)
Number of previous healthcare appointments to assess these lesions	Mean	1.36
	Median	1
	IQR	1–1
	Max	6

Overall, there was a positive sentiment regarding potential use of AIaMD in skin cancer pathways (Table 3). The majority of respondents felt confident in ‘computers’ being used to help doctors diagnose and formulate management plans (median = 70; interquartile range (IQR) = 50–95) and as a support tool for general practitioners when assessing skin lesions (median = 85; IQR = 65–100). The majority would rather have had their skin assessed by a computer than wait weeks to see an in-person dermatologist (median = 70; IQR = 50–97.5).

**Table 3:** Summary table of results from patient satisfaction of AIaMD in skin cancer pathways.

	Median	IQR
I feel confident in ‘computers’ being used to help doctors diagnose and formulate management plans	70	50–95
I think having computers assess my photographs to help guide my GP is a good way of dealing with my problem	85	65–100
I would rather have my skin assessed by a computer than wait weeks to see an in-person dermatologist	70	50–97.5
I felt comfortable having my photographs taken with a mobile phone device	95	70–100
The prospect of having my lesions assessed by a computer made me feel uncomfortable	10	0–46
I have confidence that a computer can help me and my doctor by analyzing photographs of lesions	85	60–100
I feel more confident in my diagnosis when it is made by a dermatologist compared to a computer	50	50–75
The photography service is an efficient use of my time	85	62.5–100
I found it embarrassing having my photographs taken	0	0–5
I would have preferred to see a dermatologist face to face rather than have a computer assess my lesion	50	25–80
Having a computer assess photographs of my lesion saves time in comparison to a face-to-face consultation	75	50–95
I would have preferred to have my photographs taking in my GP practice rather than in hospital	50	10–71.25
I felt the time needed to take photographs was too long	0	0–10
I would recommend the teledermatology service to friends and family.	80	50–100

Responses for most questions (9 out of 14) were comparable across the sub-groups assessed, with no significant variation in the median scores. Differences in responses were most frequently associated with the outcome of the teledermatology assessment, reaching statistical significance for four questions. Women were found to be less comfortable having photographs of their lesions taken, compared to men, while no statistically significant differences in responses were associated with respondent’s age (Table 4).

**Table 4:** Survey questions with statistically significant variation in median scores across sub-groups of respondents.

Question	Subgroup	Median	KW *p*-value
I feel confident in computers being used to help my doctor determine my diagnosis and management plan	Discharge	61.96	0.005
	Routine appointment	66.50	
	Biopsy/urgent follow up	74.61	
I think having computers assess my photographs to help guide my GP is a good way of dealing with my problem	Fitzpatrick I	79.89	0.021
	Fitzpatrick II	81.39	
	Fitzpatrick III	74.77	
	Fitzpatrick IV–VI	44.60	
I felt comfortable having my photographs taken with a mobile phone device	Discharge	75.47	0.017
	Routine appointment	86.73	
	Biopsy/urgent follow up	84.59	
	Male	85.00	0.018
	Female	80.62	
	Fitzpatrick I	84.23	0.04
	Fitzpatrick II	83.01	
	Fitzpatrick III	83.04	
	Fitzpatrick IV–VI	53.00	
I have confidence that a computer can help me and my doctor by analyzing photographs of my lesions	Discharge	71.20	0.023
	Routine appointment	79.46	
	Biopsy/urgent follow up	81.47	
I found it embarrassing having my photographs taken	Discharge	12.04	0.001
	Routine appointment	6.33	
	Biopsy/urgent follow up	7.4	
	Male	4.58	0.009
	Female	11.35	

## Discussion

AI has demonstrated potential to enhance skin cancer detection and improve efficiency in urgent cancer pathways (5), through the development of several machine learning algorithms to distinguish malignant from benign skin lesions (6). While ongoing technologies are being developed, it is paramount that patient perspectives of AI are explored in parallel, to ensure acceptability of this new technology, and to help inform successful large scale deployment into clinical pathways. Structured feedback from patients who are involved in clinical research and early deployments of AIaMD is one way in which this data can be collected.

To the best of our knowledge, this is the first comprehensive study evaluating patient perspectives of AIaMD in skin cancer pathways in the UK. Our cohort involved a large group of patients that reflect the local population who are referred on a cancer pathway, with all six Fitzpatrick skin types being represented.

Overall our study revealed a positive sentiment regarding potential use of AIaMD in skin cancer pathways. This complements a qualitative study conducted in Germany reporting 75% would recommend AI tools for skin cancer screening to family and friends, with 94% of patients expressing acceptance of the symbiosis between clinicians and AI systems (7).

The majority of our respondents felt confident in computers being used to help doctors diagnose and formulate management plans and as a support tool for general practitioners when assessing skin lesions. Importantly, our survey highlighted acceptability of AIaMD alongside clinicians as a decision-making support tool, however further assessment for stand-alone autonomous applications is required. Respondents were comfortable having their photographs taken, which is similar to other studies assessing patient acceptability of teledermatology services (8), though the differences in responses between sexes may be relevant for the wider deployment of teledermatology. Differences in responses across the Fitzptrick skin types may be influenced by the comparatively small number of responders with Fitzpatrick skin types IV–VI, and the significance of these results should be interpreted with caution.

The differences in responses associated with the outcome of the teledermatology review is interesting as those patients who were referred for a biopsy or urgent referral were consistently more willing to accept the AIaMD as part of their assessment than those who were discharged or referred for a routine appointment. This suggests patients are more amenable to new technologies being used to inform their care when they feel their condition is being more actively managed.

A common limitation of patient surveys is low participation rates, and the resultant self-selection bias with feedback missing from those patients who are unwilling or unable to participate, or those who simply forget to complete the questionnaire. The response rate for this survey was almost 40%, which is similar to response rates to online surveys elsewhere (9, 10). However, it remains possible that the results presented here are not wholly representative of the views of all patients recruited into the clinical study, and indeed the wider population of patients attending teledermatology clinics.

Further work is required to evaluate the psychological status of patients whose care involves an assessment by an AIaMD, compared to those who just attend face to face consultations. Patient feedback will continue to be important as products like DERM develop, and the clinical patient pathways in which they are deployed evolve. Further, larger studies are needed to capture patient feedback from more diverse populations, including different socio-economic groups and a wider variety of ethnicities and skin colors also focusing on acceptability of autonomous AIaMD in clinical pathways.

## Conclusion

To the best of our knowledge, this is the first comprehensive study evaluating patient perspectives of AIaMD in skin cancer pathways in the UK. Patient involvement is essential for the successful development and implementation of new technologies. Continued end-user feedback will allow refinement of services to ensure patient acceptability. This study demonstrates patient acceptability of AIaMD in both primary and secondary care settings.

## Data availability statement

The raw data supporting the conclusions of this article will be made available by the authors, without undue reservation.

## Ethics statement

The studies involving humans were approved by the West Midlands, Edgbaston Research Ethics Committee. The studies were conducted in accordance with the local legislation and institutional requirements. The patients provided their written informed consent to participate in this study.

## Author contributions

AK: Writing – original draft, Writing – review & editing. KH: Writing – review & editing. DK: Data curation, Investigation, Methodology, Resources, Validation, Writing – review & editing. PK: Formal analysis, Writing – review & editing. HM: Formal analysis, Writing – review & editing, Conceptualization, Data curation, Investigation, Methodology, Project administration, Resources, Software, Validation, Visualization. LT: Conceptualization, Methodology, Project administration, Supervision, Visualization, Writing – review & editing.
