# Supplementary figures and images for: Patient perspectives of artificial intelligence as a medical device in a skin cancer pathway
Source: Front Med (Lausanne). 2023 Nov 16;10:1259595. doi: 10.3389/fmed.2023.1259595 (PMC10693417; doi:10.3389/fmed.2023.1259595)

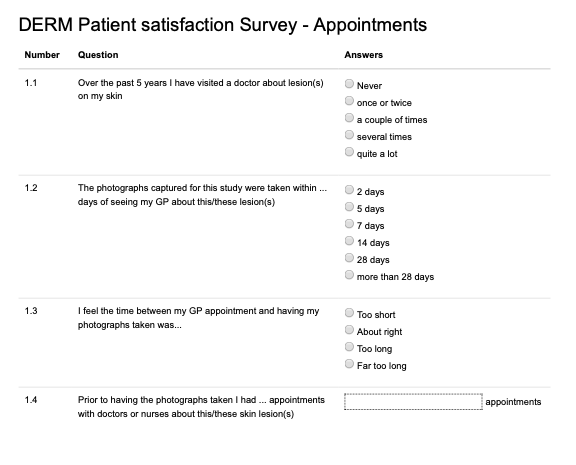

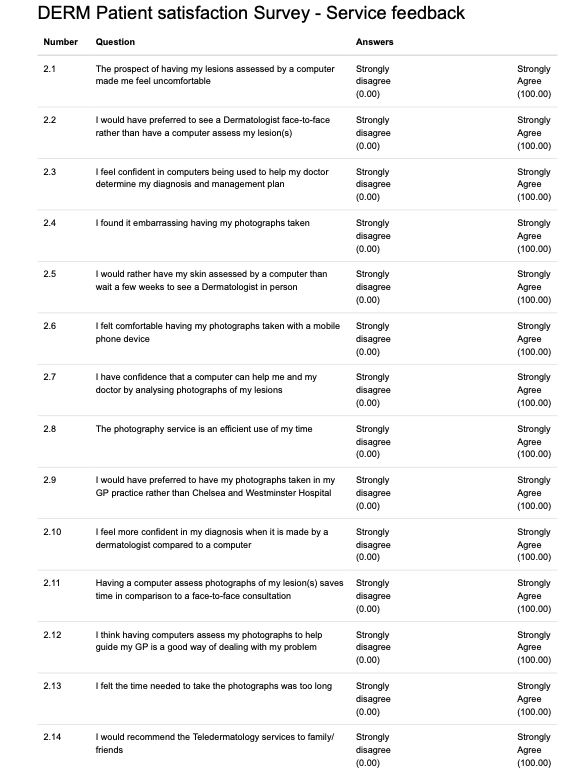


Supplementary Figure 1: patient satisfaction questionnaire

Supplement: Supplementary file 1 [file Data_Sheet_1.docx]
